# A Suppressor/Enhancer Screen in *Drosophila* Reveals a Role for Wnt-Mediated Lipid Metabolism in Primordial Germ Cell Migration

**DOI:** 10.1371/journal.pone.0026993

**Published:** 2011-11-01

**Authors:** Mark A. McElwain, Dennis C. Ko, Michael D. Gordon, Henrik Fyrst, Julie D. Saba, Roel Nusse

**Affiliations:** 1 Howard Hughes Medical Institute, Department of Developmental Biology, Stanford University School of Medicine, Stanford, California, United States of America; 2 Center for Cancer Research, Children's Hospital Oakland Research Institute, Oakland, California, United States of America; University of Dayton, United States of America

## Abstract

Wnt proteins comprise a large family of secreted ligands implicated in a wide variety of biological roles. WntD has previously been shown to inhibit the nuclear accumulation of Dorsal/NF-κB protein during embryonic dorsal/ventral patterning and the adult innate immune response, independent of the well-studied Armadillo/β-catenin pathway. In this paper, we present a novel phenotype for *WntD* mutant embryos, suggesting that this gene is involved in migration of primordial germ cells (PGC) to the embryonic gonad. Additionally, we describe a genetic suppressor/enhancer screen aimed at identifying genes required for WntD signal transduction, based on the previous observation that maternal overexpression of WntD results in lethally dorsalized embryos. Using an algorithm to narrow down our hits from the screen, we found two novel WntD signaling components: Fz4, a member of the Frizzled family, and the *Drosophila* Ceramide Kinase homolog, Dcerk. We show here that Dcerk and Dmulk (Drosophila Multi-substrate lipid kinase) redundantly mediate PGC migration. Our data are consistent with a model in which the activity of lipid phosphate phosphatases shapes a concentration gradient of ceramide-1-phosphate (C1P), the product of Dcerk, allowing proper PGC migration.

## Introduction

Signaling pathways such as those activated by the Wnt family of secreted ligands control a wide variety of biological processes. WntD (Wnt inhibitor of Dorsal, CG8458 and formerly annotated Wnt8) is a particularly interesting member of this family that is likely specific to *Drosophila* [1], [2]. WntD inhibits nuclear accumulation of the NF-κB homolog Dorsal during dorsal/ventral patterning and during the adult innate immune response. Maternal overexpression of WntD leads to lethal dorsalization of embryos, while *wntD^KO1^* mutants display a mild expansion of nuclear Dorsal protein into the anterior and posterior poles, suggesting that, *in vivo*, WntD restricts the region of Dorsal protein activity [3], [4]. Furthermore, *wntD^KO1^* mutant adults exhibit an aberrant innate immune response to bacterial infection: a subset of Dorsal target genes encoding anti-microbial peptides is more highly expressed than in WT, before and after infection. *wntD^KO1^* mutants also die earlier upon infection with pathogenic bacteria, suggesting that WntD signaling is critical in modulating the innate immune response to microbial infection [3]. This increased lethality may be due to upregulation of the TNF homolog *Eiger* and a novel gene called *Edin* in the *wntD^KO1^* mutants. Overexpression of *Edin* is sufficient to induce lethality, and *eiger* mutant flies live longer upon Salmonella infection, suggesting that higher expression of either of these genes is detrimental [5], [6].

In follow-up studies on *wntD^KO1^* mutants we discovered another role for WntD in development: regulating migration of the embryonic primordial germ cells (PGC) to the gonad. PGC, also known as pole cells, are the first cells in the *Drosophila* embryo to be specified. They begin their journey at the posterior pole, and remain outside the embryo proper upon cellularization of the embryo blastoderm [7]. During gastrulation, the PGC are carried into the embryo with the invaginating midgut epithelium. Around stage 9–10, the PGC begin to migrate across the midgut epithelium, initially dorsally along the midgut epithelium, and then sort bilaterally. As bilateral sorting proceeds, the PGC begin to be associated with mesoderm and eventually align with mesodermal somatic gonadal precursors (SGP) around stage 13. Around stage 14, the PGC and SGP coalesce to form the embryonic gonad [7], [8].

Although much is known about the cell movements and tissue interactions along the PGC migratory path, and many genes required for proper PGC guidance have been discovered, the identity of specific guidance molecules for the germ cells is yet unknown. For example, several *Drosophila* mutants in the 3-hydroxy-3-methyl-glutaryl-CoA reductase (HMGCR) pathway display defects in PGC migration, and misexpression of some of these genes is sufficient to attract PGC to ectopic tissues. These data suggest that a geranylgeranylated molecule originating in the mesoderm is either required for generation of a chemoattractant, or the geranylgeranylated molecule is secreted and acts as the attractant itself. However, this attractant has not been identified [9], [10].

Another major mechanism controlling PGC migration is through the lipid phosphate phosphatases encoded by the *Wun* and *Wun2* genes. PGC avoid tissues with endogenous or ectopic Wun or Wun2 expression, and loss of these genes results in highly disorganized PGC movement [11], [12], [13]. These studies suggest a model in which a phospholipid gradient shaped by Wun/Wun2 activity controls PGC migration, either through attraction of PGC by a phospholipid or repulsion by a non-phosphorylated lipid. It is yet unclear whether attraction or repulsion is acting upon the PGC in this model. Furthermore, *Wun* and *Wun2* encode broad-specificity lipid phosphate phosphatases, and it is unclear what the relevant substrate is in relation to PGC migration [14], [15].

Given the *wntD^KO1^* mutant phenotype in germ cell migration, we reasoned that uncovering WntD signal transduction pathway components might lend insight into the mechanism by which PGC are attracted to the gonad. Interestingly, WntD likely signals independently of the canonical Armadillo (Arm)/β-catenin pathway. Overexpression of WntD in tissues sensitive to increased levels of Arm signaling fails to produce detectable phenotypes, and germline mutations in *Daxin*, a negative regulator of Arm, do not result in embryonic dorsalization [3] [16]. In order to identify components of the WntD signal transduction pathway, we undertook a genetic screen for suppressors and enhancers of WntD-mediated embryonic dorsalization. Here, we report the results from the screen, as well as our analysis of two suppressors of WntD overexpression: *Df(1)Sxl-bt*, which removes Fz4 (CG4626), a strong candidate for the WntD receptor; and *Df(3R)e1025-14*, which removes *Drosophila* Ceramide Kinase (Dcerk, CG16708), a likely downstream effector.

Although our suppressor/enhancer screen is based on the ability of maternal WntD overexpression to lethally dorsalize embryos through inhibition of Dorsal protein activity, there are many examples of a single signaling pathway regulating multiple biological processes throughout development and homeostasis [17], [18]. It would therefore be unsurprising to find that a single pathway transducing the WntD signal can affect both dorsal/ventral patterning and PGC migration. Here, we provide evidence for ceramide kinase activity in WntD signaling and present a model in which the WntD pathway controls the production of ceramide-1-phosphate (C1P), which might be a substrate of Wun and Wun2 to attract PGC to the gonad.

## Results

### *wntD^KO1^* mutants display defects in primordial germ cell migration

**Figure 1:**
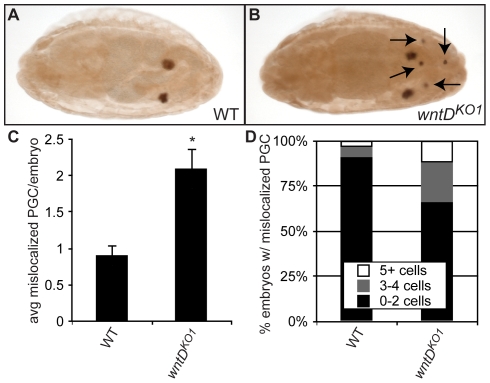
*wntD^KO1^* mutant embryos display increased numbers of mislocalized primordial germ cells. **A–B.** Embryos stained with anti-Vasa antibody to mark primordial germ cells (PGC). **A.** Representative wild-type (WT) embryo, stage 14. **B.** Representative *wntD^KO1^* embryo, stage 14. While the majority of PGC have reached the gonad and coalesced with somatic cells, arrows indicate PGC that have failed to migrate properly by this stage. **C.** Quantification of average mislocalized PGC per embryo, stage 14–16. Error bars represent standard error of the mean (SEM). Asterisk indicates P<5×10^−5^ by Student's T-test. **D.** Analysis of the number of embryos with mislocalized PGC. There is an increase in the proportion of embryos with more than 2 mislocalized cells in *wntD^KO1^* compared to WT.

We detected expression of WntD protein in tissues known to influence PGC migration, such as the invaginating midgut in stage 8 embryos (Figure S1A), and the midgut and gonad in stage 14 embryos (Figure S1B). We therefore wondered whether *wntD^KO1^* mutant embryos may exhibit a phenotype in migration of primordial germ cells (PGC) to the gonad. WT and *wntD^KO1^* mutant (maternal and zygotic) embryos were stained with antibody directed against the PGC-specific protein Vasa, and PGC that failed to migrate to the gonad by stage 14–16 were quantified (Figure 1). Indeed, we observed significantly more mislocalized PGC in *wntD^KO1^* mutants than in WT embryos. We also found a higher frequency of *wntD^KO1^* embryos with more than 2 mislocalized PGC than in WT embryos (Figure 1D).

### A suppressor/enhancer screen to identify WntD pathway components

**Figure 2:**
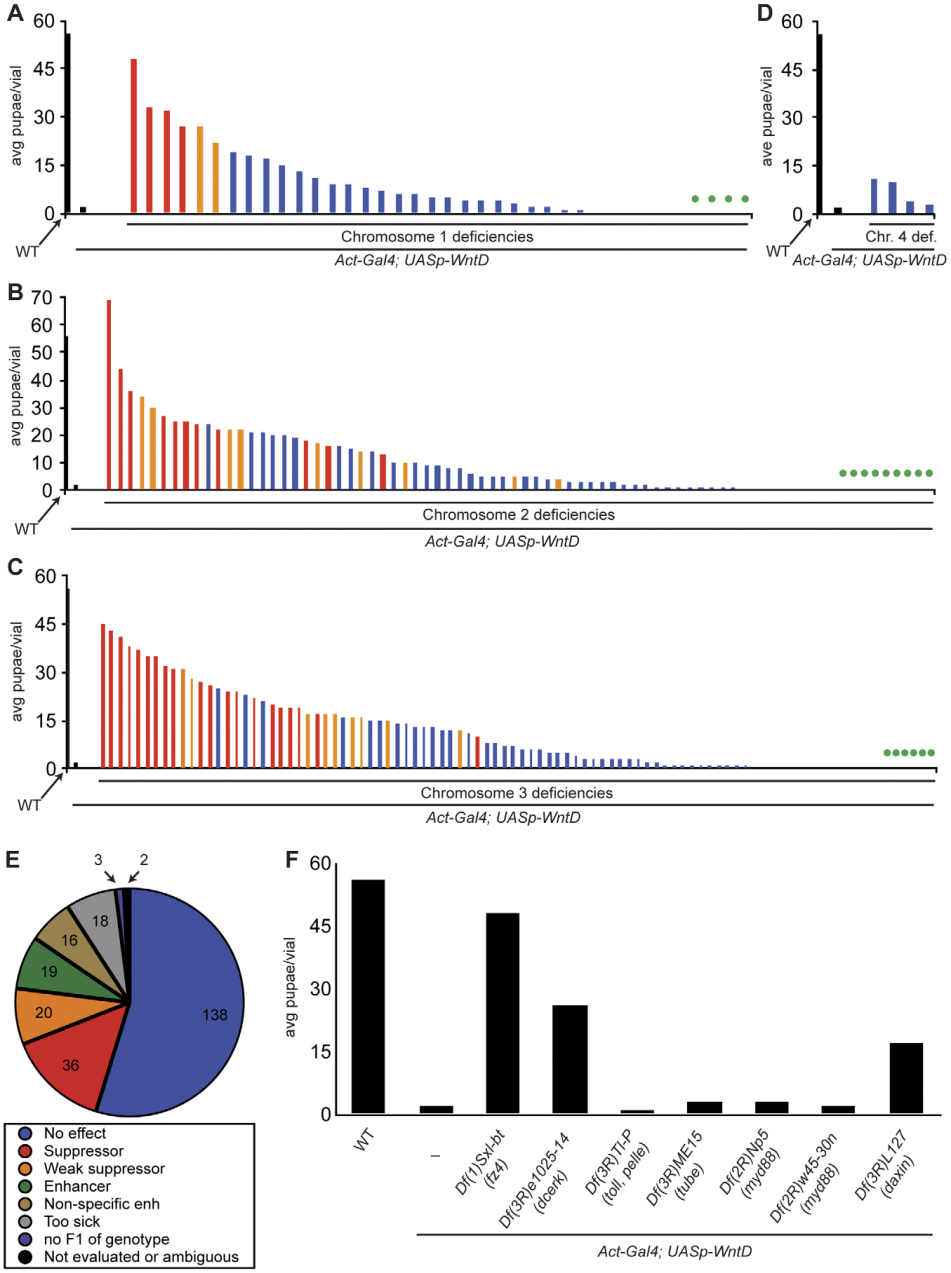
A genetic screen reveals 75 modifier deficiencies that are likely specific to the WntD pathway. **A–D.** Graphical representation of pupal counts from primary screen. Colored bars represent counts of progeny from WntD overexpressor females also carrying deficiency chromosomes. Black columns are WT and WntD overexpressor controls. Red columns represent deficiencies characterized as suppressors; orange columns represent deficiencies characterized as weak suppressors; blue columns represent deficiencies having no effect; green dots represent deficiencies characterized as enhancers. Regions with no columns and no green dot are deficiency crosses resulting in no pupae that are characterized as having no effect. A secondary screen described in Materials and Methods contributed to these final characterizations. **A.** Chromosome 1 results. **B.** Chromosome 2 results. **C.** Chromosome 3 results. **D.** Chromosome 4 results. **E.** Summary of deficiencies tested and characterizations. **F.** Selected deficiencies from the screen that remove interesting genes (*Fz4*, *Dcerk*); components of the Toll signaling pathway (*Toll*, *Tube*, *Pelle*, *Myd88*); and *Daxin*, a component of the Wnt/Armadillo signaling pathway. It is important to note that these deficiencies remove the aforementioned genes as well as dozens of other genes.

It is of interest to determine how the WntD ligand signals to influence PGC migration and other biological processes. To identify components of the WntD signaling pathway, we performed a screen for suppressors and enhancers of the lethality caused by maternal overexpression of WntD [3]. Briefly, we generated *Act-Gal4/+*; *UASp-WntD/+* females carrying deficiencies (heterozygous) from the Bloomington stock center deficiency kit and mated them to WT males (Figure S2). Progeny of *Act-Gal4/+*; *UASp-WntD/+* mothers have a low rate of survival to pupal stage, while introduction of various deficiencies to this genetic background can enhance or suppress this lethality (Figure 2). Criteria for categorizing deficiencies as enhancers or suppressors are described in materials and methods. Final categorization of deficiencies is shown in Figure 2, in which results organized by chromosome are shown graphically. Black columns indicate control crosses with WT or WntD overexpressor mothers. Red columns indicate suppression, orange columns represent weak suppression, blue columns and regions with no columns (no survival to pupal stage) represent no effect, and regions with no columns and a green dot represent enhancement. The full data set in Microsoft Excel format is available upon request.

As the lethality phenotype is a readout of signaling by the Dorsal transcription factor, suppressors and enhancers revealed by the screen might be due to deletion of genes in the Toll pathway as well as the WntD pathway. If deficiencies removing positive regulators of the Toll pathway were categorized as enhancers, this enhancing effect may be due to modification of Toll signaling and not specific to WntD signaling. However, we found that deficiencies removing the positive regulators of Toll signalling *Toll*, *Tube*, *Pelle* and *Myd88* do not enhance WntD-mediated lethality, suggesting that the modifying deficiencies revealed in the screen are likely specific to the WntD pathway (Figure 2F).

As mentioned above, previous experiments suggested no role for the β-catenin pathway in WntD signaling. To confirm this result using independent methodology, we examined deficiencies from the screen that remove regulators of Wnt-β-catenin signaling. If WntD signals via a β-catenin-dependent mechanism, deficiencies removing positive regulators of Wnt-β-catenin signaling would suppress WntD-mediated lethality and deficiencies removing inhibitors of Wnt-β-catenin signaling would enhance WntD-mediated lethality. However, we found that the deficiency *Df(3R)L127*, which removes the inhibitor of β-catenin signaling *Daxin* (as well as dozens of other genes), was categorized as a suppressor (Figure 2F). Furthermore, the *dsh^3^* null allele failed to suppress WntD overexpression (data not shown), suggesting that lethal embryonic dorsalization by WntD signaling is not mediated by Dsh or Daxin in their Wnt-β-catenin roles.

To identify genes removed by suppressor and enhancer deficiencies that are strong candidates to encode WntD pathway components, an algorithm was written that assigned a score to each gene based on several criteria. Genes with Gene Ontology (GO) terms consistent with a role in WntD signal transduction or control of Dorsal activity such “signal,” “wnt,” “dorsal,” “toll,” “immune,” “kinase,” and “phosphatase” received one point per GO term (up to three points total). Additionally, genes expressed at 2-fold different levels in *wntD^KO1^* mutant adults infected or uninfected, versus WT adults infected or uninfected received one point (“WntD/yw” and “Listeria WntD/yw” columns) [5]. Lastly, for a gene to suppress or enhance WntD signaling in this assay it must be provided maternally to the embryo. Genes expressed maternally received two points; genes not expressed maternally were penalized two points. Strong candidates identified using this method are shown in Table S1. In this paper, we further characterize two deficiencies that strongly suppress WntD overexpression: *Df(1)Sxl-bt* and *Df(3R)e1025-14* (Figure 2F).

### WntD signals through Fz4

**Figure 3:**
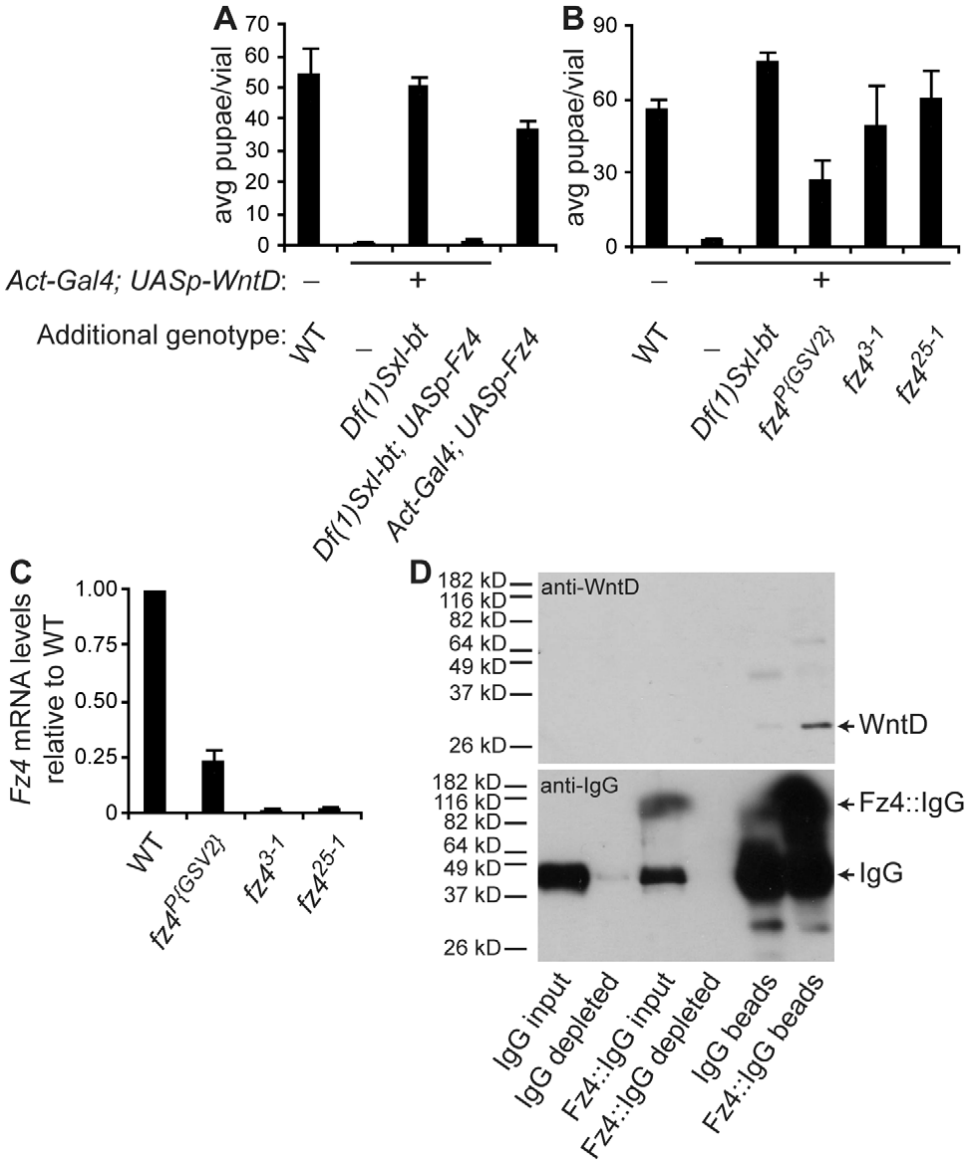
Confirmation of Fz4 as a WntD receptor. **A–B.** Error bars represent SEM. **A.** Suppression and rescue of WntD overexpression. Maternal overexpression of WntD results in low viability of progeny. Maternal overexpression of WntD in flies carrying *Df(1)Sxl-bt* results in high viability of progeny. Maternal co-overexpression of WntD and Fz4 in flies carrying *Df(1)Sxl-bt* restores low viability of progeny. Maternal overexpression of Fz4 alone has no associated lethality of progeny. **B.** Maternal overexpression of WntD results in low viability of progeny. Maternal overexpression of WntD in flies carrying *Df(1)Sxl-bt*, *fz4^P{GSV2}^*, *fz4^3-1^*, or *fz4^25-1^* results in high viability of progeny. **C.** Comparison of *Fz4* mRNA expression in WT, *fz4^P{GSV2}^*, *fz4^3-1^*, and *fz4^25-1^* adult flies. **D.** Co-immunoprecipitation of WntD and the cysteine-rich domain (CRD) of Fz4. Top panel: anti-WntD blot. WntD is highly enriched in the Fz4::IgG precipitation lane. Bottom panel: anti-IgG blot. The majority of IgG and Fz4::IgG proteins were bound to the protein G sepharose beads.

The deficiency *Df(1)Sxl-bt* is predicted to remove *Fz4* (a likely WntD signaling component) and at least 29 other genes (Flybase). In order to confirm that *Fz4*, rather than another gene removed by the deficiency, suppresses WntD overexpression, we generated *Df(1)Sxl-bt/+*; *Act-Gal4/+*; *UASp-WntD/UASp-Fz4* females and mated them to WT males, comparing survival of progeny to a WT cross, as well as WntD overexpressors with and without one copy of the *Df(1)Sxl-bt* deficiency chromosome as before. We found that Fz4 expression restores WntD-mediated lethality, suggesting that loss of *Fz4* specifically suppresses WntD overexpression. Overexpression of Fz4 alone shows no lethality (Figure 3A).

To further examine the role for Fz4 in WntD signal transduction, we wished to generate *Fz4* null alleles. We therefore performed transposase-mediated excision of P{GSV2}GS7412, a P-element inserted 217 base pairs upstream of the Fz4 start codon (Figure S3B). We recovered 55 excisions and screened for large deletions by Southern blot (a subset are shown in Figure S3C). The locations of the EcoRI sites at the *Fz4* locus used for Southern blot screening are shown in Figure S6.

Two homozygous viable and fertile excision lines, *fz4^3-1^* and *fz4^25-1^*, were analyzed further. Sequence analysis revealed that both alleles are large deletions (Figure S3B). Quantitative RTPCR showed that both alleles are null for mRNA (Figure 3C).

In order to further confirm the role for Fz4 in WntD signal transduction, we tested the novel *fz4^3-1^* and *fz4^25-1^* null alleles for the ability to dominantly suppress WntD overexpression. We compared survival of progeny from WT and WntD overexpressor females with and without one copy of each of the *Fz4* mutant alleles *Df(1)Sxl-bt*, *fz4^P{GSV2}^*, *fz4^3-1^*, and *fz4^25-1^*. We found that heterozygosity for each of these *Fz4* mutant alleles tested suppress WntD overexpression (Figure 3B).

Based on the genetic interactions between *Fz4* and *WntD*, we tested for direct binding of purified, soluble proteins by immunoprecipitation of protein G beads. We found that protein G beads highly depleted the purified Fz4::IgG fusion protein and IgG control protein from the supernatant (Figure 3D, lanes 2 and 4 of lower blot), and the Fz4::IgG bead precipitation was highly enriched for purified WntD over the IgG bead precipitation (Figure 3D, compare lanes 5 and 6 of upper blot), showing that WntD and the cysteine rich domain (CRD) of Fz4 specifically bind, in agreement with previous data showing that a membrane-bound form of WntD binds to a soluble Alkaline-Phosphatase-Fz4 fusion protein [19].

### WntD signals through Dcerk and Dmulk to control PGC migration

A second intriguing suppressor from the screen is *Df(3R)e1025-14*. This deficiency is predicted to remove *CG16708*, encoding the *Drosophila* homolog of Ceramide Kinase (hereafter referred to as *Dcerk*) [20], [21], [22], as well as 24 other genes (Flybase). Human Ceramide kinase (Cerk) catalyzes the phosphorylation of the sphingolipid ceramide to form ceramide-1-phosphate [22]. Ceramide and ceramide phosphate signaling have been implicated in a variety of biological processes, including apoptosis, inflammation, and cell proliferation [23], [24]. Because our algorithm for ranking genes identified in the screen assigned *Dcerk* a score of 3 (Table S1), and given the abundance of evidence that phospholipid metabolism controls PGC migration, we thought it likely that mutations in Dcerk could disrupt proper PGC localization to the gonad [11], [12], [14].

**Figure 4:**
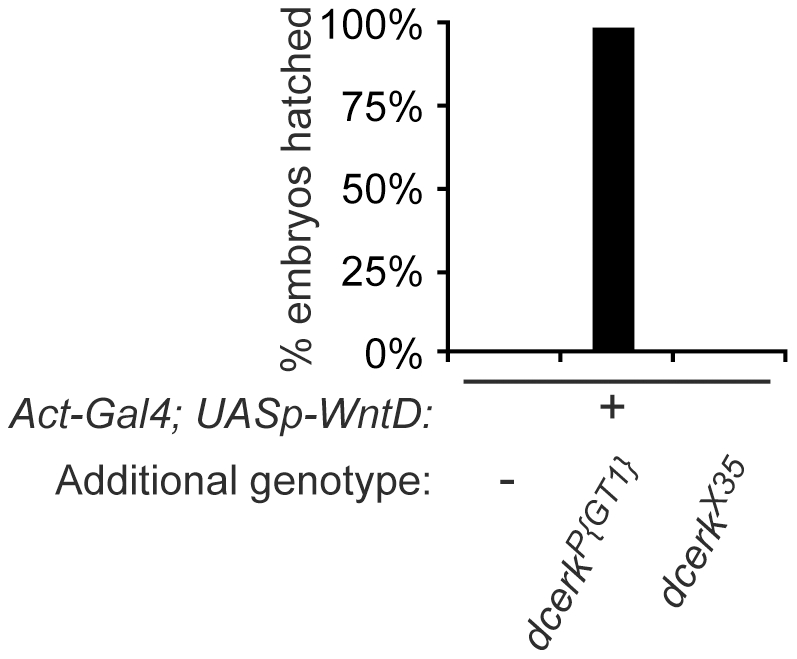
Confirming the effect of *Dcerk* mutation on WntD signaling. Comparing the survival of progeny of different maternal genotypes, as assayed by embryonic hatching. Maternal WntD overexpression results in low viability of progeny. Maternal WntD overexpression in flies also carrying a p-element insertion allele, *dcerk^P{GT1}^*, results in high viability of progeny. Maternal WntD overexpression in flies carrying a near-precise excision allele, *dcerk^X35^*, results in low viability of progeny.

To first confirm that haploinsufficiency of *Dcerk* itself confers the suppressive activity to *Df(3R)e1025-14* (Figure 2F), we obtained the homozygous viable and fertile P-element *P{GT1}BG01100* (hereafter referred to as *dcerk^P{GT1}^*), inserted in the predicted first intron of *Dcerk*. We mated *Act-Gal4/+*; *UASp-WntD/+* females, *Act-Gal4/+*; *UASp-WntD/dcerk^P{GT1}^* females and WT females to WT males and compared survival of their progeny (Figure 4). We found that the *dcerk^P{GT1}^* allele strongly suppresses WntD overexpression, similar to the original deficiency.

To confirm that the P-element insertion at the *Dcerk* locus and not a linked mutation causes suppression of WntD overexpression, we performed transposase-mediated P-element excision. By sequencing the region surrounding the *P{GT1}BG01100* insertion site we discovered that one excision allele, *dcerk^X35^*, has 38 bases of the P-element remaining at the locus (data not shown). We tested whether *dcerk^X35^* can suppress WntD overexpression by generating *Act-Gal4/+*; *UASp-WntD/dcerk^X35^* females and comparing survival of their progeny to WT females, *Act-Gal4/+*; *UASp-WntD/+* females, and *Act-Gal4/+*; *UASp-WntD/dcerk^P{GT1}^* females that had been mated to WT males. We found that, while the original *dcerk^P{GT1}^* insertion suppresses WntD overexpression, the near-precise excision *dcerk^X35^* failed to suppress, suggesting that *dcerk^X35^* retains wild-type activity, and Dcerk specifically is required for WntD signaling-mediated embryonic lethality (Figure 4).

**Figure 5:**
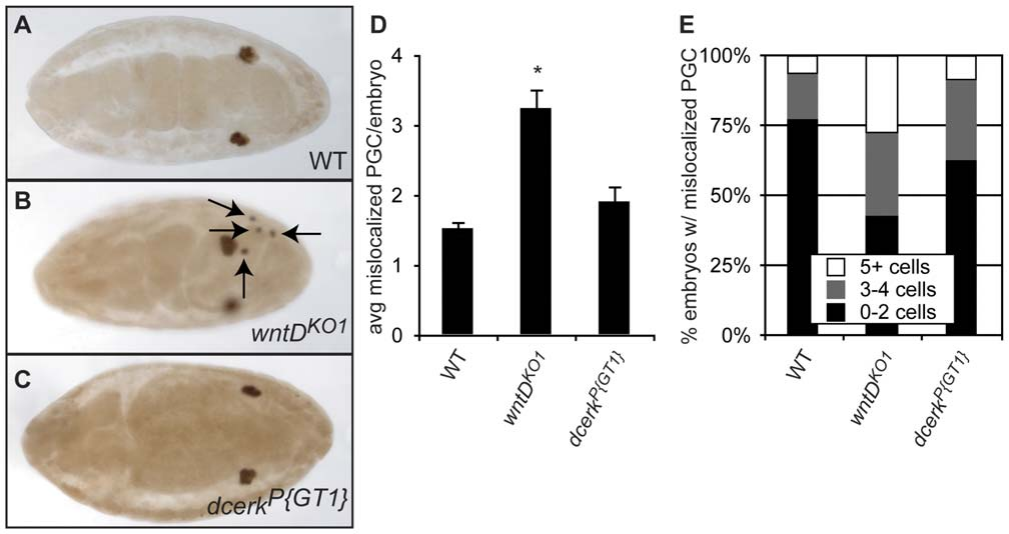
*dcerk^P{GT1}^* mutant embryos fail to phenocopy the *wntD^KO1^* PGC phenotype. **A–C.** Embryos stained with anti-Vasa antibody to mark PGC. **A.** Representative WT embryo, stage 16. **B.** Representative *wntD^KO1^* embryo, stage 16. Arrows indicate PGC that have failed to migrate properly by this stage. **C.** Representative *dcerk^P{GT1}^* embryo, stage 15. **D.** Quantification of average numbers of mislocalized PGC per embryo, stage 14–16. Error bars represent SEM. Asterisk indicates P<6×10^−10^ by Student's T-test. **E.** Analysis of the number of embryos with mislocalized PGC. There is an increase in the proportion of embryos with more than 2 mislocalized cells in *wntD^KO1^* compared to WT. *dcerk^P{GT1}^* more closely resembles WT than *wntD^KO1^*.

**Figure 6:**
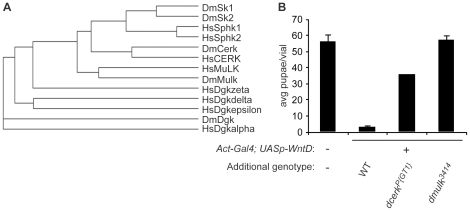
A mutation in the *D. melanogaster* Mulk homolog strongly suppresses WntD overexpression. **A.** Phylogenetic tree of *Homo sapiens* (Hs) and *Drosophila melanogaster* (Dm) lipid kinases. DmSk1 and 2, HsSphk1 and 2 are sphingosine kinases. DmCerk and HsCERK are ceramide kinases. DmMulk and HsMuLK are multi-substrate lipid kinases. Dm and HsDgk are diacylglycerol kinases. **B.** Effect of lipid kinase alleles on WntD signaling as assayed by survival to pupal stage. Maternal WntD overexpression results in low viability of progeny. Maternal WntD overexpression in flies carrying either *dcerk^P{GT1}^* or *dmulk^3414^* results in high survival of progeny. Error bars represent SEM.

When we tested homozygous *dcerk^P{GT1}^* mutant embryos for a PGC phenotype as seen in *wntD^KO1^* mutants (Figure 1), we failed to see an increase in mislocalized PGC compared to WT (Figure 5). Hypothesizing redundancy between several lipid kinases (Figure 6A), we tested mutants in the lipid kinase genes *Diacylglycerol kinase* (*dgk^f03609^*), *Sphingosine kinase 2* (*sk2^KG05894^*), *CG31873^3414^* [25], as well as four deficiencies removing *Sphingosine kinase 1* (*Sk1*) - *Df(1)BSC287*, *Df(1)BSC288*, *Df(1)BSC722*, and *Df(1)ED7067* - for the ability to dominantly suppress WntD overexpression, as before. We found that heterozygosity for either the *dcerk^P{GT1}^* allele or the *CG31873^3414^* allele strongly suppressed WntD-mediated lethality, in contrast to every other lipid kinase allele tested (Figures 6B and S4). *CG31873* encodes a homolog of mouse Multi-substrate Lipid kinase (MuLK, also known as acylglycerol kinase, Agk) reported to phosphorylate ceramide *in vitro* [26]. The *CG31873^3414^* allele is an insertion of a *piggyBac* transposon containing splice acceptors, and stop codons in all three reading frames, which generates a nonsense allele and is described in [25]. We hereafter refer to *CG31873* as *Dmulk*.

**Figure 7:**
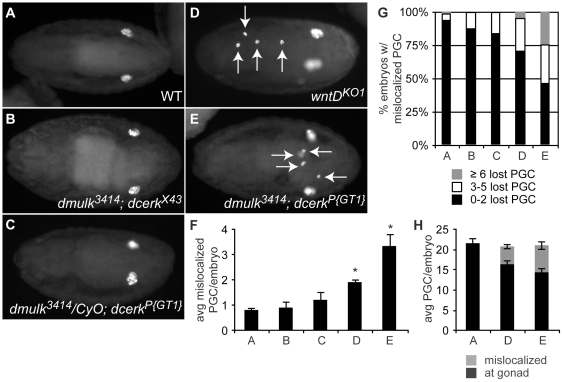
*dcerk^P{GT1}^*; *dmulk^3414^* double mutant embryos phenocopy the *wntD^KO1^* PGC phenotype. **A–E.** Representative embryos, stage 14–16, stained with anti-Vasa to visualize PGC. **A.** WT. **B.** Embryo double mutant for *dmulk^3414^*, a null allele, and *dcerk^X43^*, an excision allele expressing WT levels of *Dcerk* mRNA (data not shown). This genotype is essentially a *dmulk^3414^* alone control. **C.** Embryo heterozygous for *dmulk^3414^* and homozygous for *dcerk^P{GT1}^*. **D.** *wntD^KO1^*. **E.** Embryo double homozygous mutant *dmulk^3414^*; *dcerk^P{GT1}^*. **F–H.** Quantification of PGC mislocalization in stage 14–16 embryos of genotypes: A - WT. B - *dmulk^3414^*; *dcerk^X43^*. C - *dmulk^3414^*/*CyO*; *dcerk^P{GT1}^*. D - *wntD^KO1^*. E - *dmulk^3414^*; *dcerk^P{GT1}^*. **F.** Average numbers of mislocalized PGC per stage 14–16 embryo. Error bars represent SEM. **G.** Analysis of the number of stage 14–16 embryos with mislocalized PGC. **H.** Analysis of total numbers of PGC per stage 14–16 embryos. Mislocalized embryos are failing to migrate correctly, rather than failing to die. Error bars represent SEM.

When we labeled PGC in maternal and zygotic double mutant *dmulk^3414^*; *dcerk^P{GT1}^* embryos using the Vasa antibody, we found a significant increase in the number of mislocalized PGC in stage 14–16 mutant embryos compared to WT (Figure 7, compare A vs. E). Furthermore, neither single mutant alone displayed a significant increase in mislocalized PGC (Figures 5C and 7B), nor did *dmulk^3414^*/*CyO*; *dcerk^P{GT1}^* embryos carrying a single wild type, paternally inherited allele of *Dmulk* (Figure 7C). *dcerk^X43^* flies express wild-type levels of *Dcerk* transcript (data not shown); we therefore consider *dmulk^3414^*; *dcerk^X43^* flies to be equivalent to mutants carrying the *dmulk^3414^* allele alone (Figure 7B). Importantly, we find that *wntD^KO1^* mutant and *dmulk^3414^*; *dcerk^P{GT1}^* double mutant embryos display decreased numbers of PGC localized at the gonad when compared to WT, while total numbers of PGC within the embryo are indistinguishable from the WT, suggesting that the mislocalized cells are truly failing to migrate properly, rather than simply being “extra” cells (Figure 7H).

### Dcerk and Dmulk regulate Ceramide-1-Phosphate levels *in vivo*

**Figure 8:**
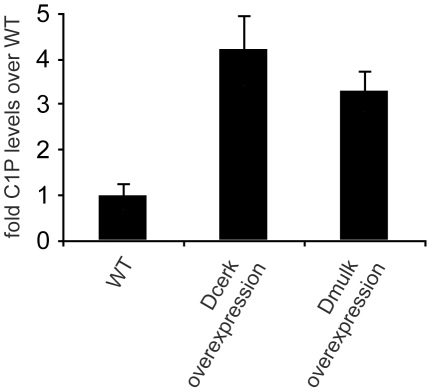
Analysis of embryonic ceramide-1-phosphate levels. HPLC-MS quantifications of C1P levels in embryos derived from WT cross, incross of *UASp-Dcerk/CyO*; *Act-Gal4/TM6B* (maternal Dcerk overexpression), and incross of *UASp-Dmulk/CyO*; *Act-Gal4/TM6B* (maternal Dmulk overexpression). C1P measurements were made in triplicate and normalized to phosphatidylcholine levels. We observed a 4.2-fold increase in C1P levels upon Dcerk overexpression and a 3.3-fold increase in C1P levels upon Dmulk overexpression.

Previous studies have shown that *Dcerk* is required for wild-type levels of ceramide-1-phosphate (C1P), suggesting that the gene encodes a bona fide ceramide kinase [21]. Our data showing that Dcerk and Dmulk redundantly control PGC migration implies that C1P, the product of ceramide phosphorylation, plays a role in guiding PGC to the gonad. This would require that Dmulk also encodes a protein with ceramide kinase activity. To test this possibility, we collected overnight egg lays from WT, *UASp-Dcerk/CyO*; *Act-Gal4/TM6B* (maternal Dcerk overexpression), and *UASp-Dmulk/CyO*; *Act-Gal4/TM6B* (maternal Dmulk overexpression) incrosses and measured C1P levels, normalized to phosphatidylcholine levels. Upon Dcerk and Dmulk overexpression, we detected a 4.2-fold and 3.3-fold increase in embryonic C1P levels over WT levels respectively, showing that both Dcerk and Dmulk are sufficient to increase C1P production (Figure 8). Full results showing the effects of Dcerk and Dmulk overexpression on C1P molecular species are shown in Figure S5. Given that the mouse homolog of Dmulk is a bona fide ceramide kinase [26], our data strongly suggest that Dmulk likely also functions as a ceramide kinase.

## Discussion

Here, we report on a suppressor/enhancer screen designed to identify members of a novel signaling pathway transducing the WntD ligand. Using the methods described above, we narrowed our initial list of 75 modifiers down to 43 genomic regions likely to encode members of the WntD pathway. We further investigated the suppressor deficiencies *Df(1)Sxl-bt* and *Df(3R)e1025-14*.

*Df(1)Sxl-bt* is predicted to remove *Fz4* and 29 other genes. Fz4 has long been a strong candidate for the WntD receptor, given previous evidence that the two proteins will bind [19]; however, until now evidence of a functional interaction has been lacking. We rescued the suppression by *Df(1)Sxl-bt* by co-overexpressing Fz4 and confirmed that mutant alleles of *Fz4* can suppress WntD overexpression, strengthening the idea that WntD signals through Fz4 *in vivo*. No other biological role has previously been ascribed to Fz4, suggesting that this may be the first Wnt-Fz interaction dedicated to activating a β-catenin-independent pathway. Indeed, the Fz4 amino acid sequence is quite different from other members of the Fz family (data not shown), suggesting an interesting signaling paradigm involving an interaction between the most divergent Wnt family member and the most divergent Fz family member.

An outstanding question is whether the receptor Fz4 contributes to PGC guidance. When we examined PGC migration in *fz4^3-1^* mutant embryos, we did not observe an increase in mislocalized cells when compared to WT (data not shown). One possible explanation for this is that, like the ceramide kinases Dcerk and Dmulk, Fz4 acts redundantly with another receptor to mediate PGC guidance. Alternatively, we cannot exclude the possibility that WntD activates different receptors to regulate D/V patterning and PGC migration. Further experimentation is required to more fully understand the biological roles of WntD/receptor interactions.

The second suppressor deficiency examined, *Df(3R)e1025-14*, is predicted to remove approximately 25 genes. Here, our algorithm allowed us to focus on a gene removed by the deficiency called *Dcerk*. We found that the P-element insertion mutant *dcerk^P{GT1}^* strongly suppressed WntD overexpression and that near-precise excision of the P-element abrogated the suppression, confirming the role of Dcerk in WntD signaling.

Given the role of Dcerk in lipid metabolism [21], [22], and the role of lipid metabolism in *Drosophila* PGC migration [11], we were interested to further explore the role of WntD-Dcerk signaling in this process. While *wntD^KO1^* mutants display a defect in migration of PGC to the gonad, *dcerk^P{GT1}^* mutants do not. However, we identified a *Drosophila* gene homologous to mouse *MuLK*, *CG31873*/*Dmulk*, which we hypothesized would compensate for loss of Dcerk because mouse Mulk can phosphorylate ceramide in vitro [26]. Indeed, embryos carrying strong loss of function alleles of both *Dmulk* and *Dcerk* display defective PGC migration. In agreement with this functional redundancy, we showed that expression of either Dcerk or Dmulk is sufficient to increase embryonic C1P levels, strongly suggesting that both Dcerk and Dmulk are ceramide kinases. Further experiments are required to directly test whether WntD signaling through these proteins regulates C1P metabolism.

These findings may shed light on previous observations regarding the influence of phospholipids on germ cell migration. Studies on *Wun* and *Wun2*, genes encoding lipid phosphate phosphatases, have generated a model in which PGC travel up a concentration gradient of a phospholipid. It is currently unclear whether PGC are attracted to the phospholipid or repelled by the non-phosphorylated version; the identity of the lipid/phospholipid is also not known [27]. Wun and Wun2 are broad-specificity phosphatases, and no lipid kinase alleles have previously been reported with PGC guidance defects in *Drosophila*. Our data are the first to indicate a role for a specific phospholipid in PGC migration and we speculate that C1P may be a relevant substrate for Wun activity in this process.

Both *Dmulk* and *Dcerk* are expressed in the midgut and hindgut starting around stage 11 of embryogenesis (BDGP in situ homepage http://insitu.fruitfly.org/cgi-bin/ex/insitu.pl). One may expect that a PGC attractant would be synthesized in the PGC target tissue. However, in this model, C1P must be synthesized and secreted from the midgut and hindgut and must diffuse readily throughout the embryo. Wun and Wun2 phosphatase activity in the gut, central nervous system, and epidermis generates a gradient of C1P such that C1P in these tissues is dephosphorylated to form ceramide, leaving the highest concentration of C1P in the lateral mesoderm, where no Wun or Wun2 is expressed. While most studies on C1P have described intracellular signaling roles for the molecule, there is evidence that C1P is readily secreted from bone marrow-derived macrophages (BMDM), providing further evidence that C1P could be used as a secreted guidance molecule in *Drosophila* [28]. We hypothesize a model in which WntD expression in the embryonic midgut stimulates the kinase activity of Dmulk and Dcerk, thereby providing C1P as a PGC chemoattractant.

One possible weakness in this model is that, if all ceramide kinase activity were removed from the embryo, it might be expected that no C1P would be generated. If C1P were the phospholipid guiding PGC to the gonad, a very strong phenotype would be observed, as is the case in strong *wun*, *wun2* mutants [29]. However, the phenotype in *dmulk^3414^*; *dcerk^P{GT1}^* mutants is weak in comparison (Figure 7). This may be explained by the fact that the *Dcerk* allele used in this study is not a complete null; adult *dcerk^P{GT1}^* flies express approximately 20% of wild-type levels of *Dcerk* mRNA (data not shown). Furthermore, in *dmulk^3414^*; *dcerk^P{GT1}^* double mutant adults, C1P levels are reduced only 2-fold compared to WT (data not shown). If this is also true in embryos, it likely explains why this comparatively weak phenotype is observed. It is possible that true *dmulk*; *dcerk* double null embryos would have completely disrupted PGC migration.

It is also possible that C1P can be generated via a mechanism besides ceramide phosphorylation, and true *dmulk*; *dcerk* double null embryos would still have significant levels of C1P. Indeed, when *Cerk* null mice were generated, it was found that, while *cerk* ^−/−^ BMDM have no detectable ceramide kinase activity, cellular levels of C1P were approximately 25% those of wild-type cells [28]. These data suggest there is indeed a ceramide kinase-independent source of C1P in mice; it is likely that this is also the case in other organisms. There are currently no data suggesting a mechanism for this in mouse or *Drosophila*; however, some arachnid, bacteria and fungus species express a Sphingomyelinase D (SmaseD) protein capable of metabolizing sphingomyelin directly into C1P [30]. While a BLAST search of the *Drosophila* genome reveals no clear SmaseD homologs, we believe it possible that this type of Smase activity may be encoded by genes not apparently related evolutionarily to SmaseD. In this case, even in the complete absence of ceramide phosphorylating activity, a significant amount of C1P may be available for the generation of a gradient by Wun and Wun2 activity. This hypothetical ceramide-kinase-independent source of C1P would also explain the comparatively weak phenotype observed in *wntD^KO1^* embryos.

It is interesting to note that studies have shown that Wun2 can dephosphorylate phosphatidic acid (PA) and lysophosphatidic acid (LPA), but not C1P [14], [15]. However, mammalian homologs of Wun can dephosphorylate C1P, as well as S1P, PA and LPA, raising the possibility that Wun and Wun2 can as well [15], [31]. If this is true, it is unclear why studies have failed to show Wun or Wun2 activity against C1P. However, given the fact that the majority of *Drosophila* sphingolipids contain a 14-carbon backbone, while mammalian and commercially-synthesized sphingolipids contain an 18-carbon backbone, we believe it to be likely that *Drosophila* proteins have decreased affinity for commonly available sphingolipid reagents [32]. Furthermore, we cannot exclude the possibility that Wun activity generates a gradient of multiple different phospholipids used in PGC guidance. It will be interesting to see in the future if further experimentation can address this question.

While vertebrates do not express a WntD ortholog, studies of *Drosophila* germ cell migration may lend insight into mechanisms of vertebrate cell migration. Granado *et al.* suggest that C1P induces macrophage migration, although the effect appears to be much weaker than that induced by S1P [33]. Further experimentation in both mammalian and *Drosophila* systems may reveal similarities in how a gradient of C1P regulates migration of a variety of cell types.

We have presented here the results of an unbiased genetic screen aimed at identifying genes in a novel signaling pathway that transduces the WntD signal. We present the first evidence of a functional interaction between WntD and its receptor Fz4, and identify a novel downstream effector, Dcerk. We further hypothesized that the same transduction mechanism is used to influence dorsal/ventral patterning, the adult innate immune response, and PGC migration. Indeed, we present evidence that two ceramide kinases redundantly mediate the effect of WntD on both dorsal/ventral patterning and PGC migration. Further experimentation will determine whether this pathway also impacts the immune response. Interestingly, our unbiased screen led us to a line of experimentation suggesting a role for ceramide-1-phosphate in PGC migration, the first evidence for a specific phospholipid in this process. Further studies should directly test whether C1P represents the long-unknown substrate for Wun and Wun2 in PGC migration.

## Materials and Methods

### Embryonic Antibody staining

Embryos were removed from agar collection caps with a paintbrush and dechorionated in 50% bleach for 5 minutes, followed by rinsing in cold water. Embryos were then fixed in 4% paraformaldehyde and heptane, devitellinized by methanol/heptane, and stained with antibodies using standard protocols. Antibodies and dilutions used were: rabbit anti-Vasa 1∶1000–1∶2000 (gift, Yuh Nung Jan) [34]; rabbit anti-WntD 1∶500 [3]; mouse anti-β-galactosidase 1∶200 (Promega).

### Screen crosses and general husbandry

Flies were maintained under standard conditions unless otherwise noted. WT flies are *yw*.

For the suppressor/enhancer screen, *Act-Gal4*/*CyO*; *UASp-WntD*/*Tm6B* males were mated to virgin females heterozygous for a single deficiency from the Bloomington deficiency kit (or WT virgins as control). After 5 days at 25°C the crosses were shifted to 28°C. Upon eclosion, 5–6 *Act-Gal4*; *UASp-WntD* virgin females carrying one deficiency chromosome were mated to WT males and progeny surviving to pupae were quantified after 10–11 days.

For the first pass of analysis, when crosses gave rise to more than zero but fewer than 25 progeny that survive to pupal stage the deficiencies were categorized as having no effect. When crosses gave rise to more than 25 progeny that reached pupal stage, the deficiencies were initially categorized as suppressors. Exceptions were made for several crosses resulting in fewer than 25 pupae when many larvae were observed; these deficiencies were also categorized as suppressors. When crosses resulted in no churning of the media, indicating zero survival to larval stage, the deficiencies were categorized as enhancers. Full results are available in Microsoft Excel format.

Putative suppressors were subjected to a secondary, more quantitative screen in which the above crosses were repeated and embryonic hatching to first-instar larvae was quantified. Crosses giving rise to embryos with a hatch rate from 1–5% were categorized as having no effect. Crosses with hatch rates from 6–19% were categorized as weak suppressors. Crosses with hatch rates greater than or equal to 20% were categorized as strong suppressors.

To rule out the possibility that significant lethality indicating enhancement of WntD overexpression could be due to reduced viability associated with the deficiency itself, rather than enhancement of WntD signaling, females carrying the deficiency plus either the *Act-Gal4* or *UASp-WntD* transgenes, but not both (and therefore not overexpressing WntD), were mated to WT males and survival of progeny was assayed. Deficiencies conferring significant lethality were categorized as “non-specific enhancers” and were excluded from further analysis.

### WntD suppression by hatch assay

To assay suppression of WntD overexpression by embryonic hatch assay, crosses were performed as described above, but matings between *Act-Gal4*; *UASp-WntD* virgins carrying a deficiency and WT males were placed in crossing cages. After 24 hours of embryo collection, collection caps were removed and embryos were aged for 24 hours. Embryos hatching to first-instar larvae were then quantified.

### Generation of *Fz4* null alleles

Females homozygous for *P{GSV2}fz4^GS7412^* were mated to males carrying a Δ2–3 transposase insertion. *P{GSV2}fz4^GS7412^*; Δ2–3 males were then mated to virgin females carrying the *Binsinscy* balancer. White-eyed excision/*Binsinscy* progeny were mated to *Binsinscy* males to establish stable stocks.

### Generation of *Dcerk* excision alleles

Flies carrying the *dcerk^P{GT1}^* allele and a Δ2–3 transposase transgene were mated to TM3 balancer flies and the progeny were screened for loss of the *w^+^* marker.

### Southern Blot

For genomic DNA isolation, 15 adult females of each genotype (WT, *P{GSV2}fz4^GS7412^*, *P{GSV2}fz4^GS7412^*/*Binsinscy*, and homozygous or *Binsinscy*-balanced *P{GSV2}fz4^GS7412^* excisions) were crushed with a Kontes pestle in 75 µL lysis buffer (100 mM Tris pH 8.8, 100 mM EDTA, 1% SDS). An additional 225 µL buffer was added and samples were further homogenized. Samples were then incubated at 72°C for 30 min. and 66 µL KOAc (5 M) was added. Samples were incubated for 30 min. on ice, followed by centrifugation at 20,800×g for 15 min. at 4°C. The supernatant was transferred to another tube and centrifuged again. The supernatant was again transferred and one volume isopropanol was added. The samples were centrifuged again for 5 min at room temperature to precipitate genomic DNA. Pellets were washed with 70% ethanol and centrifuged once more. The pellets were briefly allowed to air-dry and resuspended in 105 µL TE, and incubated for several minutes at 37°C to dissolve.

The entire genomic DNA preparation was digested overnight at 37°C with EcoRI and subjected to electrophoresis through duplicate 0.7% agarose gels. DNA was transferred to Hybond XL membranes (GE Healthcare) overnight by alkaline transfer in denaturation buffer (0.5 M NaOH, 1.5 M NaCl). Following transfer, membranes were incubated for 30 min. in neutralization buffer (0.5 M Tris pH 6.0, 1.5 M NaCl), washed twice in 2xSSC, and pre-hybridized in Rapid-Hyb buffer (GE Healthcare) for 30 min at 65°C.

Probes were generated from genomic DNA by PCR using primers “sthrn probeC Fw” (5′- gatctatagcatatctcacca-3′) and “sthrn probeC Re” (5′- tcaccgtggacggactgtcca-3′). Probes were labeled with 32P alpha-dCTP (Perkin Elmer) using the Rediprime II Random Prime Labeling System (GE Healthcare) according to the manufacturer's instructions. Membranes were incubated overnight at 65°C with probes in Rapid-Hyb buffer, and then washed for 20 min. at room temperature in 2xSSC, 0.1% SDS; 15 min. at 65°C in 0.5xSSC, 0.1%SDS; and 15 min. at 65°C in 0.1xSSC, 0.1% SDS. Hyperfilm MP (GE Healthcare) was exposed to membranes at −80°C.

Locations of EcoRI sites with respect to the *P{GSV2}* insertion and *Fz4* start codon are indicated in Figure 6.

### PCR analysis of *Fz4* mutant alleles

Pfu Ultra II Fusion HS DNA Polymerase (VWR) was used to PCR amplify the *fz4^3-1^* and *fz4^25-1^* loci. Primers Fz4excscreenFw1 (5′-gctgacttgcctcttgtagag-3′) and Fz4excscreenRe1 (5′-gacgagttgcttggtgagatatg-3′) were used to amplify the *fz4^3-1^* locus. Primers Fz4PscreenFw1 (5′- ggaactggcactggggttgcc-3′) and Fz4PscreenRv1 (5′- gatcgttttcgggttaatccc-3′) were used to amplify the *fz4^25-1^* locus. PCR products were submitted for sequencing by Elim Biopharm (Hayward, CA), revealing that *fz4^3-1^* is a major deletion of 2013 bp and a minor deletion of 18 bp located 9 base pairs upstream of the major deletion and that *fz4^25-1^* is a 1042 bp deletion in the promoter region (Figure S3B).

### Generation of transgenic lines

Primers NOT1CG16708 (5′-aaggaaaaaagcggccgcatgacgcagaccaagcagcca-3′) and Xba1CG16708 (5′-agtctagactagtccttgcagcagcagta-3′) were used to amplify the *Dcerk* coding region. Primers KpndMuLKFwd (5′-gaggtaccatgaattatttaagagtaattc-3′) and XbadMuLKRev-stop (5′-attctagatcaacaaaaaactttaatagc-3′) were used to amplify the *CG31873*/*Dmulk* coding region. PCR products were purified, digested, and ligated into UASp vector. UASp expression vectors were co-injected with a transposase expression vector into *yw* embryos using a pulled-glass needle.

### Quantitative RT-PCR

Primers dCERKRTPCRFwd2 (5′-gcatctcccgttacagtca-3′) and dCERKRTPCRRev2 (5′-aattcccttgtgcgatatacctcta-3′) were used to detect *Dcerk* transcripts. Primers fz4RTfwd1 (5′-aaacgggcaacgatcac-3′) and fz4RTrev1 (5′-gactccaagggccatgt-3′) were used to detect *Fz4* transcripts. Primers RpS15AFwd2 (5′-ctctggcggcatcatgga-3′) and RpS15ARev1 (5′-gttggttgcatggtcggtga-3′) were used to detect *RpS15A* transcripts.

To purify mRNA, 6 adult females of each genotype were homogenized in 150 µL Trizol (Invitrogen) using a Kontes pestle. An additional 150 µL Trizol was added and flies were homogenized some more. Thereafter, the standard Trizol protocol was followed.

cDNA was synthesized using a Thermoscript kit (Invitrogen) using included oligo-dT primer and standard protocols. Quantitative PCR was performed using Sybr Green PCR master mix (Applied Biosystems) on a Step One Plus thermocycler (Applied Biosystems). *Fz4* mRNA levels were normalized against *RpS15A* mRNA levels.

### Ceramide-1-phosphate quantifications

Prior to lipid extractions, *Drosophila* embryos were collected on agar caps using standard methods, dechorionated in 50% bleach, and washed several times in methanol. Embryos were stored at −20°C in methanol until lipid extraction.

Ceramide-1-phosphate was extracted from *Drosophila* adults and embryos following a previously described method [35]. C8-ceramide-1-phosphate (Cayman Chemicals, Ann Arbor, Michigan) was added as an internal standard. Lipid extracts were transferred onto a C18 reverse phase column (Luna 50×2 mm 3 µ, Phenomenex, Torrance, CA) equilibrated in 50% solvent A (methanol∶water∶acetic acid 50∶49∶1) and 50% solvent B (methanol∶acetic acid 99∶1). Both solvents contained 5 mM ammonium acetate. Ceramide-1-phosphate molecular species were eluted at a flow rate of 0.3 ml/min using a gradient from 50–100% solvent B in solvent A in 15 min. Structural conformation was obtained by positive electrospray ionization tandem mass spectrometry (ESI(+)-MS/MS). Lipids were identified based on their specific precursor and product ion pair and quantified using multiple monitoring as previously described [36]. MRM transitions monitored were: 562.5/208.5 (d14:1/16:0), 590.5/208.5 (d14:1/18:0), 618.5/208.5 (d14:1/20:0), 646.5/208.5 (d14:1/22:0), 674.5:208.5 (d14:1/22:0).

### Co-immunoprecipitation

WntD protein was purified as previously described; however, the blue Sepharose fractions contained only WntD and bovine serum albumin and were considered pure [37]. Further purification steps were omitted.

IgG and Fz4::IgG proteins were purified as previously described [38]. The Fz4::IgG construct is an Ig-tagged version of the cysteine rich domain (CRD) of Fz4, which is the high-affinity Wnt-binding cassette of Fz family members.

Protein G-coupled sepharose beads were incubated with equal masses of purified IgG or Fz4::IgG protein overnight at 4°C. Beads were washed with cold PBS and incubated with equal amounts of purified WntD protein overnight at 4°C. Beads were again washed with cold PBS. After washing, sample buffer was added to beads and incubated 5 min at room temperature. All samples were then subjected to SDS-PAGE, followed by western blot by standard protocols. Antibody dilutions: Rabbit anti-WntD 1∶1000, AP-conjugated goat anti-Rabbit 1∶20000 (Santa Cruz Biotechnology, Santa Cruz, CA), AP-conjugated goat anti-human 1∶20000 (Bio-Rad, Hercules, CA).

## Supporting Information

Figure S1 **Tissue-specific WntD antibody staining.** WT embryos stained with WntD antibody. **A.** Stage 8 embryo. Expression is observed in invaginating midgut. **B.** Stage 14 embryo. Expression is observed in midgut (arrow) and gonads (arrowheads).
(PDF)

Figure S2 **Genetic crossing scheme for WntD suppressor/enhancer screen.** **A–C.** Deficiency stock virgin females were mated to *Act-Gal4/CyO*; *UASp-WntD/TM6B* males and virgin female progeny with no balancers were selected. These females, overexpressing WntD in the germline and carrying a deficiency, were mated to WT males and survival of progeny was assayed. **A.** Scheme for X-chromosome deficiencies. **B.** Scheme for 2^nd^ chromosome deficiencies. **C.** Scheme for 3^rd^ chromosome deficiencies.
(PDF)

Figure S3 **Imprecise excision of P-element in the Fz4 promoter region results in likely null alleles.** **A.** Crossing scheme to excise *fz4^P{GSV2}^* P-element and recover potential *Fz4* mutant alleles. **B.** Diagram of *Fz4* locus. Grey boxes: 5′ and 3′ UTR. Black boxes: exons. *fz4^P{GSV2}^* is inserted 64 bp upstream of the *Fz4* transcriptional start site and 217 bp upstream of the start codon. White boxes indicate extent of major deletions of *fz4^3-1^* (2013 bp deletion) and *fz4^25-1^* (1042 bp deletion) alleles. **C.** Southern blot of *Fz4* excision alleles. Lane 1: WT. Lane 2: *fz4^P{GSV2}^/Binsinscy*. Lane 3: Homozygous *fz4^P{GSV2}^*. Lane 7: *fz4^3-1^*. Band is shifted above expected size of 1.7 kb for intact P-element, and below expected size of 7.8 kb for WT locus or a precise excision, indicating a partial deletion of *Fz4*. Lane 42: *fz4^25-1^*. Band (visible above background in right-hand side of lane) appears to be shifted slightly below expected size for WT locus, indicating partial deletion of *Fz4*. All remaining lanes are uncharacterized excision alleles and are homozygous, except for lanes 13 and 51, which are balanced over *Binsinscy*.
(PDF)

Figure S4 **Mutations in Ceramide kinases, but no other lipid kinases, can suppress WntD overexpression.** Maternal WntD overexpression results in low survival of progeny. Maternal WntD overexpression in flies carrying *dcerk^P{GT1}^* results in high survival of progeny. Maternal WntD overexpression in flies carrying *dgk^f03609^*, *sk2^KG05894^*, or any of four different deficiencies predicted to remove *Sk1* results in low survival of progeny.
(PDF)

Figure S5 **Dcerk and Dmulk display differential specificity for ceramide molecular species.** **A.** Maternal Dcerk and Dmulk overexpression results in increased embryonic C1P levels compared to WT. Error bars represent SEM. Asterisks represent P<0.05 by Student's T-test compared to WT. Dcerk overexpressor embryos are from a UASp-Dcerk/CyO; Act-Gal4/TM6B incross. Dmulk overexpressor embryos are from a UASp-Dmulk/CyO; Act-Gal4/TM6B incross. **B.** Measurements of embryonic C1P level normalized to phosphatidylcholine levels, +/− standard deviation (SD).
(PDF)

Figure S6 **Locations of EcoRI sites at the** ***Fz4*** **locus.** EcoRI sites within the *P{GSV2}* P-element and flanking the Southern blot probe (indicated by blue bar) at the *Fz4* locus imply predicted bands of 7.8 kb for a wild-type locus or precise P-element excision; 1.7 kb for a locus with intact P-element, or in which part of the P-element has been excised while the internal EcoRI site remains inserted; 7.8–12.5 kb for a partial P-element excision in which the internal EcoRI has been excised; or <7.8 kb for a deletion of the entire P-element plus surrounding *Fz4* genomic DNA.
(PDF)

Table S1 Strong WntD pathway candidates from screen. High-scoring candidates were narrowed by effects of overlapping deficiencies, by virtue of association with GO terms consistent with signaling pathways, significant change in expression by microarray [5], and by maternal expression. “Total genes” indicates the number of genes in the region encompassed by the deficiency, while “Remaining candidate genes” indicates the number of genes after taking into account the phenotypes of other overlapping deficiencies from the kit as well as smaller overlapping deficiencies assayed after identification of the candidate regions. The “WntD/yw” column indicates the fold difference in the gene's expression level in uninfected *wntD^KO1^* mutant adults compared to uninfected *yw* adults, and “Listeria WntD/yw” indicates fold difference in the gene's expression level in *wntD^KO1^* mutant adults compared to *yw* adults after injection with *Listeria monocytogenes* [5]. Genes further characterized in this article are indicated in bold.
(PDF)
