# Supplementary figures and images for: A Suppressor/Enhancer Screen in Drosophila Reveals a Role for Wnt-Mediated Lipid Metabolism in Primordial Germ Cell Migration
Source: PLoS One. 2011 Nov 1;6(11):e26993. doi: 10.1371/journal.pone.0026993 (PMC3206050; doi:10.1371/journal.pone.0026993)

Figure S1

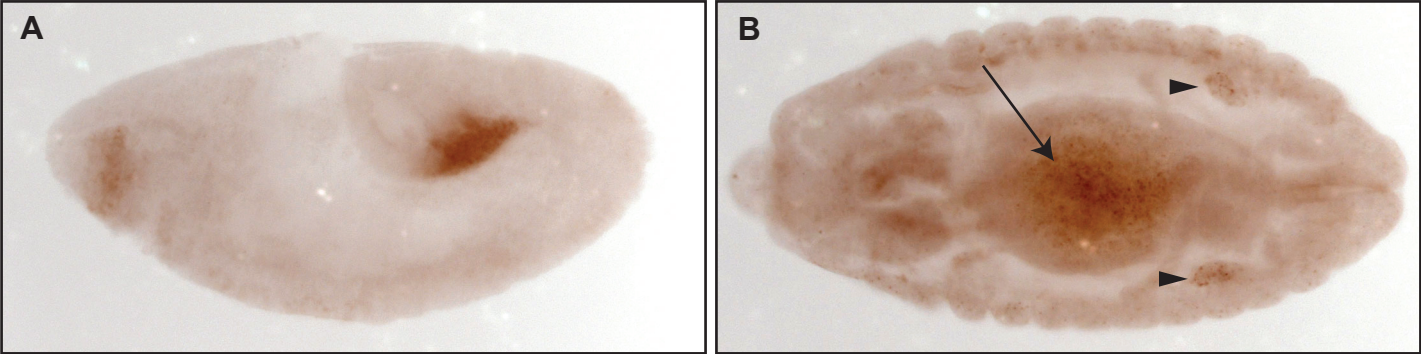

Supplement: Figure S1 — Tissue-specific WntD antibody staining. WT embryos stained with WntD antibody. A. Stage 8 embryo. Expression is observed in invaginating midgut. B. Stage 14 embryo. Expression is observed in midgut (arrow) and gonads (arrowheads). (PDF) [file pone.0026993.s001.pdf]

**Figure S3**

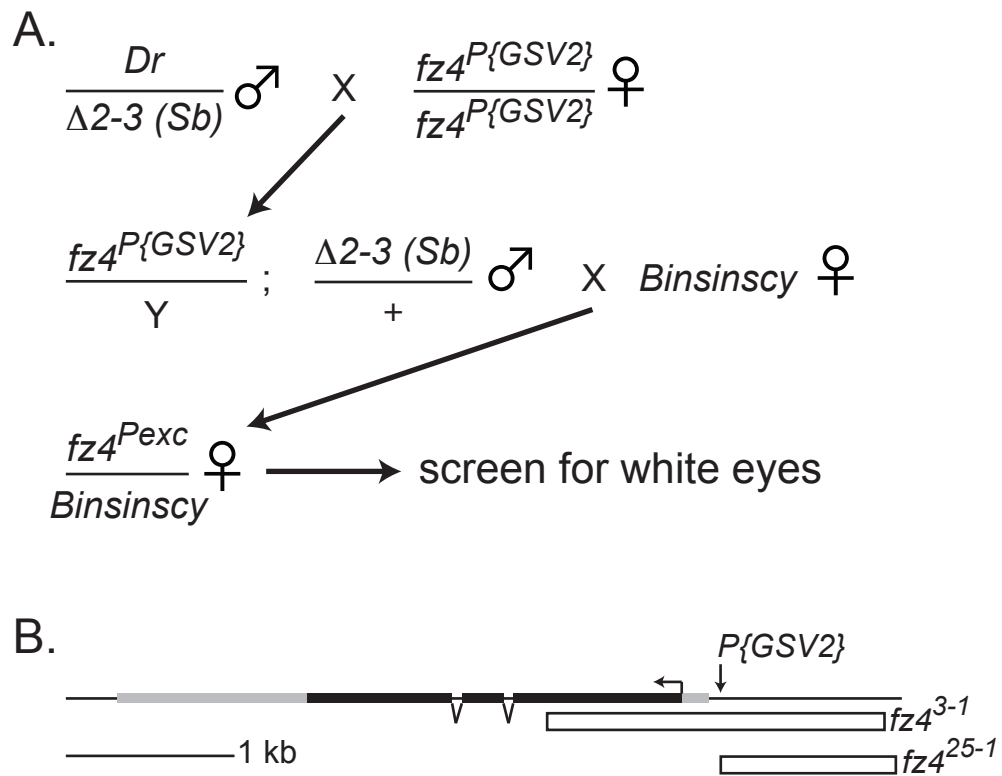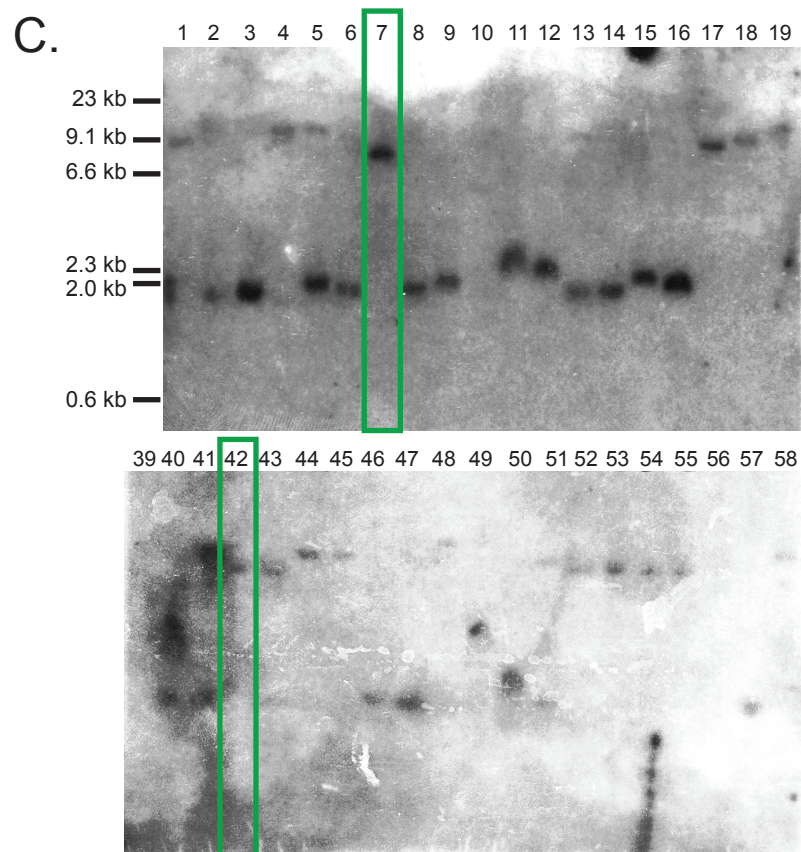

Supplement: Figure S3 — Imprecise excision of P-element in the Fz4 promoter region results in likely null alleles. A. Crossing scheme to excise fz4P{GSV2} P-element and recover potential Fz4 mutant alleles. B. Diagram of Fz4 locus. Grey boxes: 5′ and 3′ UTR. Black boxes: exons. fz4P{GSV2} is inserted 64 bp upstream of the Fz4 transcriptional start site and 217 bp upstream of the start codon. White boxes indicate extent of major deletions of fz43-1 (2013 bp deletion) and fz425-1 (1042 bp deletion) alleles. C. Southern blot of Fz4 excision alleles. Lane 1: WT. Lane 2: fz4P{GSV2}/Binsinscy. Lane 3: Homozygous fz4P{GSV2}. Lane 7: fz43-1. Band is shifted above expected size of 1.7 kb for intact P-element, and below expected size of 7.8 kb for WT locus or a precise excision, indicating a partial deletion of Fz4. Lane 42: fz425-1. Band (visible above background in right-hand side of lane) appears to be shifted slightly below expected size for WT locus, indicating partial deletion of Fz4. All remaining lanes are uncharacterized excision alleles and are homozygous, except for lanes 13 and 51, which are balanced over Binsinscy. (PDF) [file pone.0026993.s003.pdf]

Figure S4

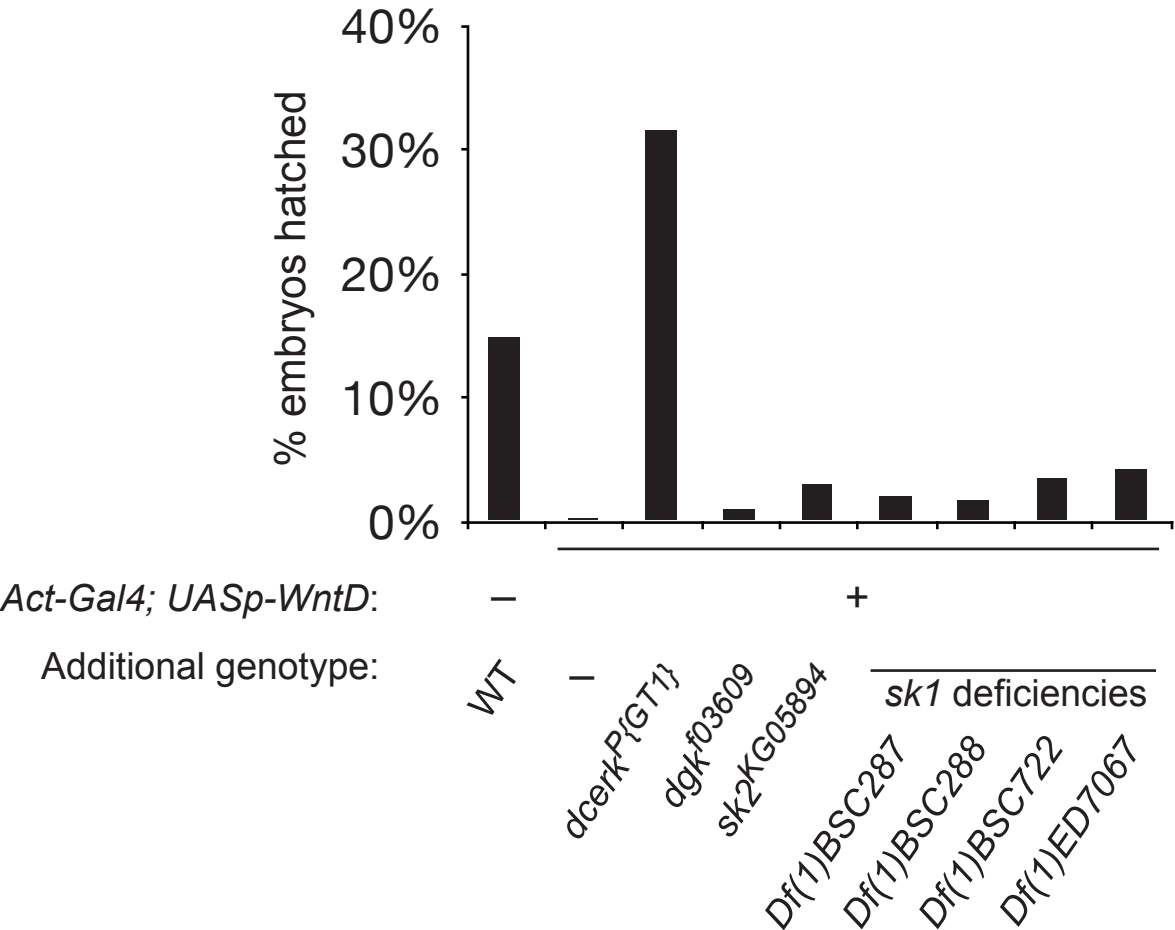

Supplement: Figure S4 — Mutations in Ceramide kinases, but no other lipid kinases, can suppress WntD overexpression. Maternal WntD overexpression results in low survival of progeny. Maternal WntD overexpression in flies carrying dcerkP{GT1} results in high survival of progeny. Maternal WntD overexpression in flies carrying dgkf03609, sk2KG05894, or any of four different deficiencies predicted to remove Sk1 results in low survival of progeny. (PDF) [file pone.0026993.s004.pdf]

Figure S6

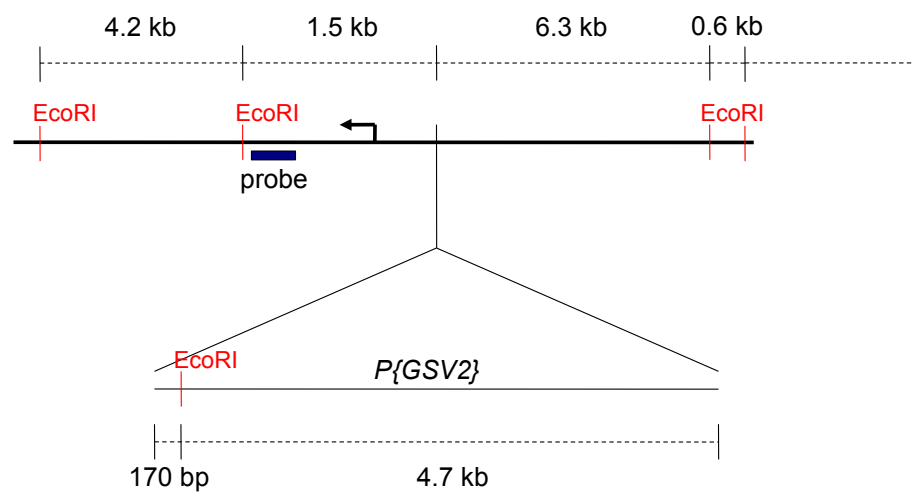

Supplement: Figure S6 — Locations of EcoRI sites at the Fz4 locus. EcoRI sites within the P{GSV2} P-element and flanking the Southern blot probe (indicated by blue bar) at the Fz4 locus imply predicted bands of 7.8 kb for a wild-type locus or precise P-element excision; 1.7 kb for a locus with intact P-element, or in which part of the P-element has been excised while the internal EcoRI site remains inserted; 7.8–12.5 kb for a partial P-element excision in which the internal EcoRI has been excised; or <7.8 kb for a deletion of the entire P-element plus surrounding Fz4 genomic DNA. (PDF) [file pone.0026993.s006.pdf]
