# Supplementary material for: A Suppressor/Enhancer Screen in Drosophila Reveals a Role for Wnt-Mediated Lipid Metabolism in Primordial Germ Cell Migration
Source: PLoS One. 2011 Nov 1;6(11):e26993. doi: 10.1371/journal.pone.0026993 (PMC3206050; doi:10.1371/journal.pone.0026993)

Figure S2

A.

X Chromosome deficiencies

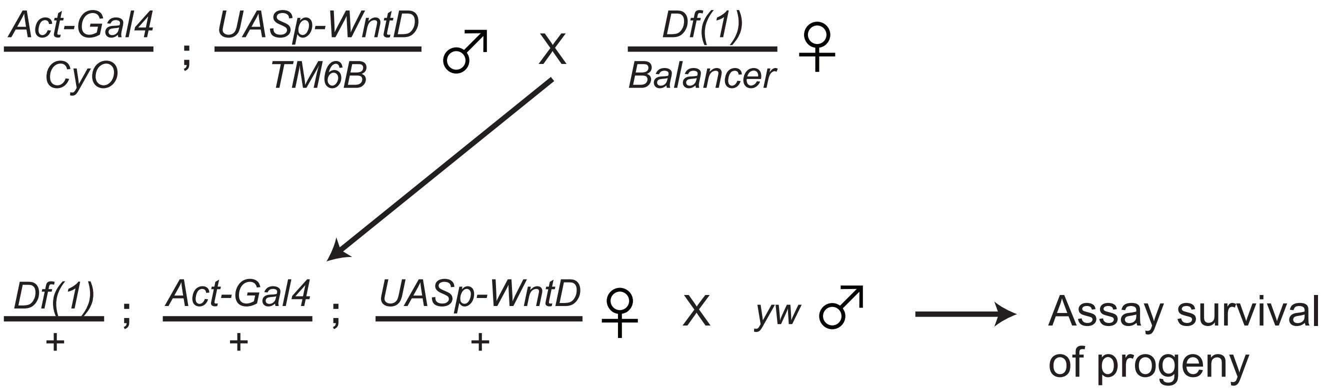

B.

2nd Chromosome deficiencies

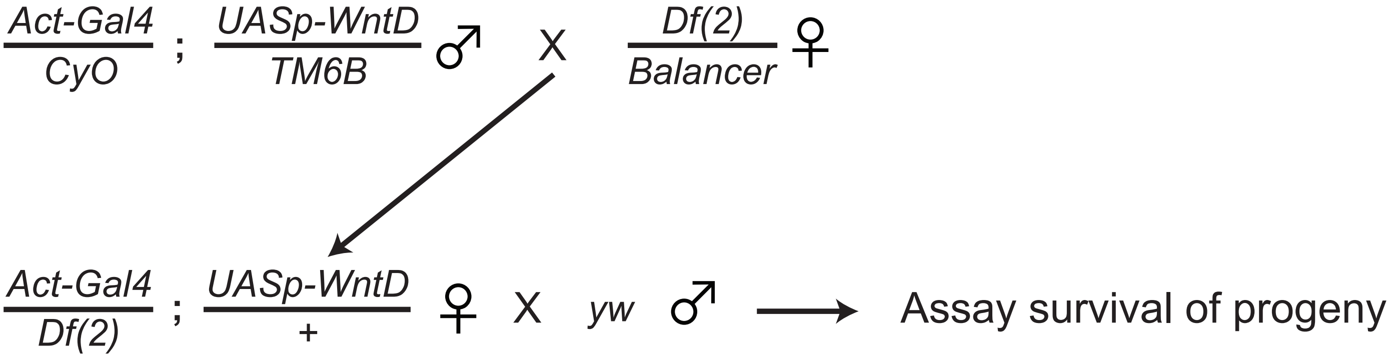

C.

3rd Chromosome deficiencies

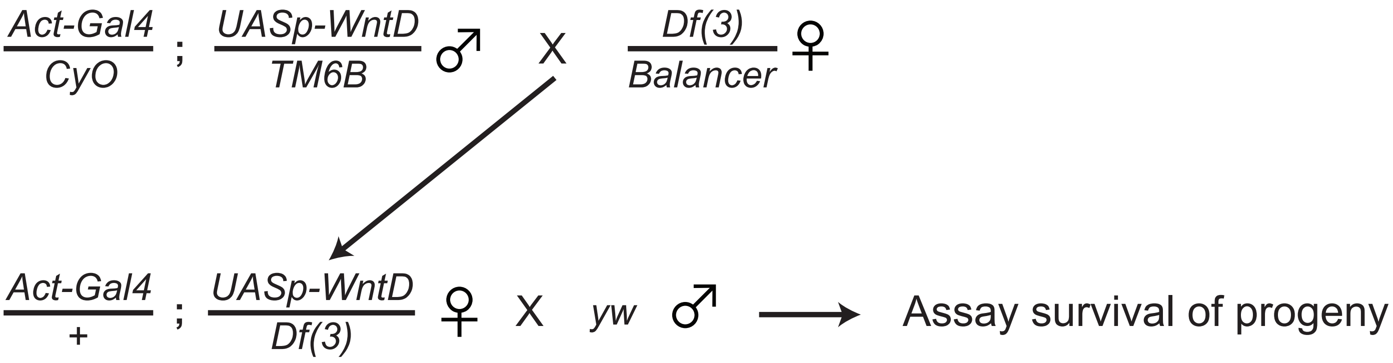

Supplement: Figure S2 — Genetic crossing scheme for WntD suppressor/enhancer screen. A–C. Deficiency stock virgin females were mated to Act-Gal4/CyO; UASp-WntD/TM6B males and virgin female progeny with no balancers were selected. These females, overexpressing WntD in the germline and carrying a deficiency, were mated to WT males and survival of progeny was assayed. A. Scheme for X-chromosome deficiencies. B. Scheme for 2nd chromosome deficiencies. C. Scheme for 3rd chromosome deficiencies. (PDF) [file pone.0026993.s002.pdf]
