# Supplementary material for: A Suppressor/Enhancer Screen in Drosophila Reveals a Role for Wnt-Mediated Lipid Metabolism in Primordial Germ Cell Migration
Source: PLoS One. 2011 Nov 1;6(11):e26993. doi: 10.1371/journal.pone.0026993 (PMC3206050; doi:10.1371/journal.pone.0026993)
Supplement: Figure S5 — Dcerk and Dmulk display differential specificity for ceramide molecular species. A. Maternal Dcerk and Dmulk overexpression results in increased embryonic C1P levels compared to WT. Error bars represent SEM. Asterisks represent P<0.05 by Student's T-test compared to WT. Dcerk overexpressor embryos are from a UASp-Dcerk/CyO; Act-Gal4/TM6B incross. Dmulk overexpressor embryos are from a UASp-Dmulk/CyO; Act-Gal4/TM6B incross. B. Measurements of embryonic C1P level normalized to phosphatidylcholine levels, +/− standard deviation (SD). (PDF) [file pone.0026993.s005.pdf]

Figure S5

A

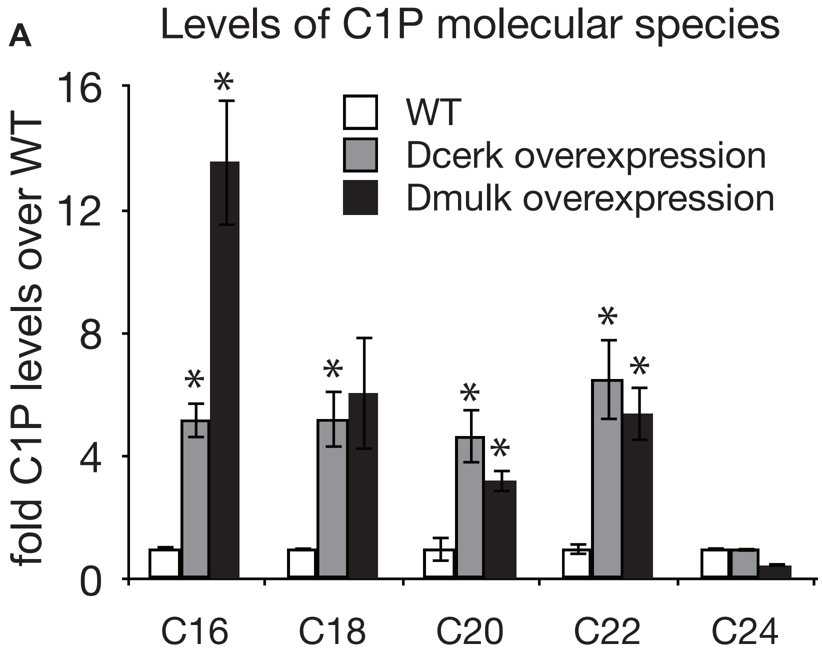

B

genotype	pmol ceramide-1-phosphate/µg phosphatidylcholine +/- SD					
yw	0.02524 +/- 0.0007160	0.05109 +/- 0.009371	0.7393 +/- 0.5783	0.2267 +/- 0.09009	0.2827 +/- 0.04987	1.325 +/- 0.7270
Dcerk overexpression	0.1310 +/- 0.02767	0.2660 +/- 0.08483	3.437 +/- 1.166	1.474 +/- 0.5320	0.2816 +/- 0.05813	5.590 +/- 1.837
Dmulk overexpression	0.3421 +/- 0.09225	0.3091 +/- 0.1655	2.372 +/- 0.5156	1.223 +/- 0.3638	0.1298 +/- 0.01522	4.376 +/- 1.066
molecular species:	C16	C18	C20	C22	C24	Total
