# Supplementary material for: A Suppressor/Enhancer Screen in Drosophila Reveals a Role for Wnt-Mediated Lipid Metabolism in Primordial Germ Cell Migration
Source: PLoS One. 2011 Nov 1;6(11):e26993. doi: 10.1371/journal.pone.0026993 (PMC3206050; doi:10.1371/journal.pone.0026993)
Supplement: Table S1 — Strong WntD pathway candidates from screen. High-scoring candidates were narrowed by effects of overlapping deficiencies, by virtue of association with GO terms consistent with signaling pathways, significant change in expression by microarray [5], and by maternal expression. “Total genes” indicates the number of genes in the region encompassed by the deficiency, while “Remaining candidate genes” indicates the number of genes after taking into account the phenotypes of other overlapping deficiencies from the kit as well as smaller overlapping deficiencies assayed after identification of the candidate regions. The “WntD/yw” column indicates the fold difference in the gene's expression level in uninfected wntDKO1 mutant adults compared to uninfected yw adults, and “Listeria WntD/yw” indicates fold difference in the gene's expression level in wntDKO1 mutant adults compared to yw adults after injection with Listeria monocytogenes [5]. Genes further characterized in this article are indicated in bold. (PDF) [file pone.0026993.s007.pdf]

| Region | Deficiency (stock number) | Effect | Total genes | Remaining candidate genes | Top Candidate Gene(s) | Found keywords | WntD/yw | Listeria WntD/yw | Maternal? | Score |
|---|---|---|---|---|---|---|---|---|---|---|
| *Chromosome 1* | | | | | | | | | | |
| 1 | 1329 | Suppressor | 145 | 16 | CG2995 | | 1.04 | 1.34 | yes | 2 |
| | | | | | cin | | 1.01 | 0.99 | yes | 2 |
| | | | | | y | | 0 | 0 | yes | 2 |
| 2 | 945, 946 | Enhancer | 50 | 10 | none score above 0 | | | | | |
| 3 | 3196, 5280 | Suppressor | 28 | 5 | fz4 | wnt signal | 1.15 | 0.94 | no data | 2 |
| | | | | | CG4615 | | 1.02 | 0.82 | yes | 2 |
| | | | | | CG4617 | | 0.95 | 0.91 | yes | 2 |
| 4 | 727 | Enhancer | 115 | 52 | NFAT | signal | 0.88 | 1.81 | yes | 3 |
| 5 | 1039 | Suppressor | 44 | 18 | CG9053 | | 1.04 | 1.01 | yes | 2 |
| | | | | | CG5599 | | 1.27 | 1.22 | yes | 2 |
| | | | | | Cyp4s3 | | 2.06 | 2.12 | no data | 2 |
| | | | | | RpL37a | | 0.91 | 0.97 | yes | 2 |
| 6 | 5272 | Enhancer | 62 | 48 | rok | signal dorsal kinase | 1.05 | 1.19 | no data | 3 |
| | | | | | if | dorsal | 1.09 | 1.21 | yes | 3 |
| | | | | | CG4789 | signal | 1.06 | 1.13 | yes | 3 |
| 7 | 972 | Suppressor | 250 | 31 | Tak1 | signal dorsal immune kinase | 0.96 | 1.01 | yes | 5 |
| | | | | | cactin | signal dorsal | 1.09 | 1 | yes | 4 |
| *Chromosome 2* | | | | | | | | | | |
| 8 | 3638 | Suppressor | 51 | 8 | spen | wnt signal | 0.99 | 0.91 | yes | 4 |
| 9 | 6283 | Enhancer | 34 | 34 | ebi | signal | 1.15 | 1.03 | yes | 3 |
| | | | | | crq | immune | 0.92 | 0.85 | yes | 3 |
| 10 | 6608 | Suppressor | 2 | 1 | CG4297 | | 1.17 | 1.08 | no | -2 |
| 11 | 1567 | Suppressor | 54 | 33 | Mad | signal dorsal | 1 | 1.19 | yes | 4 |
| 12 | 6875, 90 | Enhancer | 31 | 11 | CG17257 | | 1.05 | 1.04 | yes | 2 |
| 13 | 8674 | Suppressor | 8 | 4 | CG5828 | kinase | 1.13 | 0.98 | yes | 3 |
| 14 | 2414 | Suppressor | 80 | 6 | CG10158 | | 1.08 | 1.06 | yes | 2 |
| | | | | | SA | | 1.09 | 1.02 | yes | 2 |
| | | | | | Pcp | | 0 | 0 | yes | 2 |
| | | | | | CG31908 | | 1.03 | 1 | yes | 2 |
| 15 | 5869 | Suppressor | 50 | 9 | none score above 0, no in situ data for any | | | | | |
| 16 | 567 | Suppressor | 206 | 206 | Nak | signal kinase | 1.01 | 0.73 | yes | 4 |
| 17 | 7145 | Suppressor | 35 | 21 | 7 genes score 2 | | | | | |
| 18 | 7445 | Enhancer | 89 | 43 | 11 genes core 2 | | | | | |
| 19 | 7441 | Enhancer | 59 | 14 | Ngp | signal | 1.02 | 1.03 | yes | 3 |
| 20 | 6866 | Suppressor | 44 | 19 | CG7097 | signal kinase | 1 | 0.99 | yes | 4 |
| | | | | | Fak56D | signal kinase | 1.22 | 1.01 | yes | 4 |
| 21 | 7896 | Enhancer | 25 | 9 | 5 genes score 2 | | | | | |
| 22 | 7273 | Suppressor | 119 | 28 | 10 genes score 2 | | | | | |

| Region | Deficiency (stock number) | Effect | Total genes | Remaining candidate genes | Top Candidate Gene(s) | Found keywords | WntD/yw | Listeria WntD/yw | Maternal? | Score |
|---|---|---|---|---|---|---|---|---|---|---|
| 23 | 2604 | Suppressor | 62 | 9 | CG3608 | kinase | 1.16 | 1.31 | yes | 3 |
| *Chromosome 3* | | | | | | | | | | |
| 24 | 600 | Suppressor | 92 | 5 | R | signal dorsal | 0.98 | 0.97 | yes | 4 |
| 25 | 6755 | Suppressor | 57 | 20 | CG11486 | kinase | 0.87 | 0.99 | yes | 3 |
| 26 | 6964 | Suppressor | 16 | 14 | RhoGEF4 | signal | 0 | 0 | yes | 3 |
| | | | | | Srp19 | signal | 1.3 | 1.21 | yes | 3 |
| 27 | 1420 | Enhancer | 117 | 29 | msk | signal kinase | 0 | 0 | yes | 4 |
| 28 | 5877 | Suppressor | 75 | 75 | msk | signal kinase | 0 | 0 | yes | 4 |
| 29 | 7079, 997 | Suppressor | 140 | 2 | CG4080 | | 2.13 | 1.7 | yes | 3 |
| 30 | 2612 | Suppressor | 153 | 6 | Adk1 | kinase | 1.13 | 1.15 | no data | 1 |
| | | | | | CG5626 | kinase | 1.3 | 1.3 | no data | 1 |
| 31 | 3126, 6551 | Suppressor | 153 | 4 | Toll-6 | signal toll kinase | 0 | 0 | yes | 5 |
| 32 | 2990 | Suppressor | 81 | 68 | 5 genes score 2 | | | | | |
| 33 | 6754 | Suppressor | 16 | 1 | fz2 | wnt signal | 1.17 | 1.09 | no data | 2 |
| 34 | 2052 | Enhancer | 78 | 18 | 5 genes score 2 | | | | | |
| 35 | 5878 | Enhancer | 44 | 14 | CG12983 | | 1.2 | 1.17 | yes | 2 |
| 36 | 4429, 4430 | Suppressor | 93 | 19 | Pc | | 0.87 | 0.98 | yes | 2 |
| 37 | 5694 | Suppressor | 22 | 1 | CG16708 | kinase | 0.82 | 0.76 | yes | 3 |
| 38 | 1931 | Enhancer | 116 | 3 | PpD3 | phosphatase | 0.92 | 1.01 | yes | 3 |
| 39 | 1920 | Suppressor | 66 | 48 | CSN5 | signal dorsal | 1.08 | 1.05 | yes | 4 |
| 40 | 8583 | Enhancer | 37 | 6 | cenB1A | signal | 0.98 | 0.85 | yes | 3 |
| | | | | | cnc | dorsal | 0.94 | 1.02 | yes | 3 |
| 41 | 2585, 7675 | Suppressor | 25 | 7 | Rab7 | signal | 1.02 | 1.08 | no data | 1 |
| 42 | 2363 | Suppressor | 37 | 10 | CG6364 | kinase | 1.16 | 1.02 | yes | 3 |
| | | | | | Pp1alpha-96A | phosphatase | 0 | 0 | yes | 3 |
| 43 | 3547 | Suppressor | 130 | 12 | 8 genes score 2 | | | | | |
